# Homer1 gene products orchestrate Ca^2+^-permeable AMPA receptor distribution and LTP expression

**DOI:** 10.3389/fnsyn.2012.00004

**Published:** 2012-09-27

**Authors:** Andrei Rozov, Aleksandar R. Zivkovic, Martin K. Schwarz

**Affiliations:** ^1^IZN and Department of Clinical Neurobiology, University HospitalHeidelberg, Germany; ^2^Division of Neuroscience, Medical Research Institute Ninewells Hospital and Medical School, Dundee UniversityDundee, UK; ^3^Department of Molecular Neurobiology, Max-Planck Institute for Medical ResearchHeidelberg, Germany

**Keywords:** homer, AMPA receptors, LTP, transgenic mouse models, receptor trafficking

## Abstract

We studied the role of Homer1 gene products on the presence of synaptic Ca^2+^-permeable AMPA receptors (AMPARs) and long-term potentiation (LTP) generation in hippocampal CA1 pyramidal neurons, using mice either lacking all Homer1 isoforms (Homer1 KO) or overexpressing the immediate early gene (IEG) product Homer1a (H1aTG). We found that Homer1 KO caused a significant redistribution of the AMPAR subunit GluA2 from the dendritic compartment to the soma. Furthermore, deletion of Homer1 enhanced the AMPAR-mediated component of glutamatergic currents at Schaffer collateral synapses as demonstrated by increased AMPA/NMDA current ratios. Meanwhile, LTP generation appeared to be unaffected. Conversely, sustained overexpression of Homer1a strongly reduced AMPA/NMDA current ratios and polyamine sensitivity of synaptic AMPAR, indicating that the proportion of synaptic GluA2-containing AMPAR increased relative to WT. LTP maintenance was abolished in H1aTG. Notably, overexpression of Homer1a in Homer1 KO or GluA2 KO mice did not affect LTP expression, suggesting activity-dependent interaction between Homer1a and long Homer1 isoforms with GluA2-containing AMPAR. Thus, Homer1a is essential for the activity-dependent regulation of excitatory synaptic transmission.

## Introduction

Synaptic plasticity and memory consolidation are dynamically regulated by the activation of immediate early gene (IEG) expression, which can directly modulate postsynaptic signaling mechanisms in excitatory neurons (Lanahan and Worley, 1998). Homer1a, a prominent IEG, is generated by endogenous synaptic activity inducing conversion of intronic to exonic sequence during transcription of the Homer1 gene (Brakeman et al., 1997; Bottai et al., 2002). Long Homer1 isoforms contain an N-terminal EVH domain, whereby they associate with proline-rich motives in key signaling constituents in postsynaptic spines, and a C-terminal coiled-coil domain plus leuzine zipper for multimerization (Tu et al., 1998, 1999; Beneken et al., 2000). In contrast, the IEG-form Homer1a lacks the C-terminal multimerization sequences and thus competes with long Homer1 isoforms in binding postsynaptic signaling proteins (Xiao et al., 2000; Duncan et al., 2005; Celikel et al., 2007). Thus, in postsynaptic spines of excitatory neurons different Homer isoforms can modulate the function of a variety of synaptic components, including ionotropic and metabotropic glutamate receptors, transient receptor potential channels, shank scaffolding proteins, and endoplasmatic reticulum Ca^2+^ release channels (Kammermeier et al., 2000; De Bartolomeis and Iasevoli, 2003; Duncan et al., 2005). In neurons of WT mice, Homer1a is barely detectable, but is drastically increased during learning new behavioral tasks and epileptic seizures (Vazdarjanova et al., 2002; Altar et al., 2004; Cavarsan et al., 2012). Thus, Homer1a might play an important role in memory formation under physiological condition and additionally might have neuroprotective functions during pathological events. Indeed, transgenic overexpression of Homer1a in hippocampal CA1 neurons abolishes the maintenance of CA3-CA1 long-term potentiation (LTP) and impairs spatial working memory (Celikel et al., 2007). It has been shown that the initial phase of LTP is associated with an increase in the GluA2-lacking population of synaptic AMPA receptors (AMPARs) at CA1 Schaffer collateral synapses, either due to functional modification or insertion from intracellular pools (Plant et al., 2006; Guire et al., 2008; Moult et al., 2010). In contrast, other groups could not obtain evidence for the involvement of Ca^2+^-permeable AMPARs in the initial phase of LTP (Adesnik and Nicoll, 2007; Gray et al., 2007).

We found that sustained overexpression of Homer1a leads to significantly reduced AMPA/NMDA current ratios, accompanied by the loss of polyamine and philanthotoxin-433 (PhTx-433) sensitivity of synaptic AMPARs during single-pulse stimulation. We conclude that the contribution of GluA2-lacking channels to synaptic responses in CA1 pyramidal neurons is greatly reduced by overexpression of Homer1a. In turn, loss of synaptic GluA2-lacking channels abolished LTP generation. The induction of LTP in these synapses resulted in a transient potentiation reaching baseline ~20 min after induction. This effect was dependent on GluA2-containing synaptic AMPARs and long Homer1 isoforms, since LTP maintenance was not affected by Homer1a overexpression in hippocampal neurons lacking either the GluA2 subunit, or Homer1 proteins. Collectively, we suggest that Homer1a is essential for activity-dependent redistribution of synaptic AMPARs, which might regulate the level and duration of LTP.

## Materials and methods

### Generation of Homer1 knock-out mice

The Homer1 targeting construct was cloned from a 14 kb SV29 mouse genomic DNA insert from a lambda phage library harboring exons 2, 3, and 4 of the Homer1 gene. A SV40 NEO cassette containing a single loxP site and two Frt sites was inserted ~700 bp downstream of exon 2. The second loxP element was inserted ~200 bp upstream of exon 2. In addition, we generated 5 silent mutations in exon 2 in order to check the transcription level of the targeted allele in comparison to the WT allele. After linearization the resulting targeting construct was electroporated into R1 embryonic stem cells. Homologous recombination was confirmed by southern blotting. After blastocyst injection chimeras were mated to C57Bl/6 mice to produce Homer1 heterozygotes. To remove the NEO cassette mice were mated to Flp transgenic mice resulting in conditional Homer1 knock-out mice. To generate a functional, global Homer1 KO, heterozygotes were bred with CMV-Cre transgenic mice. All mice we used in this study were backcrossed at least for 10 generations into C57Bl/6 background. The recombination events were confirmed by southern blot and routinely monitored by PCR genoyping (Yuan et al., 2003).

### Semiquantitative evaluation of GluA2 distribution in dorsal hippocampus

The pixel intensity of hippocampal subfields (*x* = 300 μm, *y* = 120 μm) of pyramidal-and dendritic layers in GluA2 fluorescently stained pictures of dorsal hippocampus from Homer1 KO (*n* = 3) and WT littermates (*n* = 3) were determined using FIJI (ImageJ) software. To measure the redistribution of GluA2 from dendritic to pyramidal layer in Homer1 KO mice we divided the mean pixel intensity measured in the pyramidal cell layer by the mean pixel intensity of the dendritic layer. A ratio above 1 indicated higher GluA2 levels in the pyramidal cell layer compared to the dendritic layer. A ratio below 1 indicated higher GluA2 levels in the dendritic layer compared to the pyramidal cell layer.

### Immunohistochemistry

Adult male mice (~8 weeks) were transcardially perfused with 4% PFA. Brain sections (70 μm) were pre-incubated in 5% normal goat serum (NGS; Sigma, St. Louis, MO) for 2 h at room temperature and transferred to monoclonal Glu2A (Chemicon, 1:5000), monoclonal NeuN (Millipore, 1:500) and polyclonal GluN1 (Millipore, 1:500) antibody solutions containing 0.5% Triton X-100 and 1% NGS in phosphate-buffered saline (PBS). Sections were incubated in primary antibody solution for 48 h at 4°C, washed 10 min in PBS, and incubated in fluorescently labeled (FITC, TRIC) secondary antibody (goat anti-mouse, goat anti-rabbit IgG 1:500; Vector; Burlingame, CA) for 2 h at room temperature. After washing in PBS (10 min), the sections were mounted on Superfrost glass slides (Menzel; Braunschweig, Germany), dried, dehydrated, and cover-slipped with Xylene for confocal microscopy.

### Lentivirus production and injections

Lentiviruses were produced according to (Dittgen et al., 2004). Briefly, human embryonic kidney 293FT cells (Invitrogen; Carlsbad, CA) were transfected by using the calcium-phosphate method with three plasmids: expression vector and two helper, 8.9 and vesicular stomatitis virus G protein vectors at 1, 7.5, and 5.5 mg of DNA per 10-cm plate. After 48 h, the supernatants of four plates were pooled, spun at 780 g for 5 min, filtered at a 0.45-μm pore size, spun at 83,000 g for 1.5 h, and the pellet was resuspended in 100 ml of PBS. Homer1a-Venus (H1aV) [WT or mutant (W24A)] was expressed from a lentiviral vector under the control of a-CaMKII promoter, FCK(1.3)W (Dittgen et al., 2004). For stereotaxic injections of the viruses, we used standard surgical techniques (Dittgen et al., 2004). Briefly, animals were anesthetized with ketamine hydrochloride (90 mg/kg, i.p.)/xylazine (5 mg/kg, i.p.) for surgical anesthesia. All pressure points and the skin incision were infused with Lidocain (1% Lidocainhydrochlorid-1 H_2_0). The stereotaxic coordinates were (from Bregma; in mm): AP, −2.6; ML, 3.6; depth, 4.0–2.5 (with 5 injections at 0.3 *z*-step).

### Electrophysiology

Transverse hippocampal 250 mm slices were prepared from the brains of 42–56 day-old WT, *Homer1 KO, Homer1aTG* and *GluA2^−/−^* mice, killed by decapitation. The slicing chamber contained an oxygenated ice-cold solution (modified from (Dugue et al., 2005) composed of (in mM): K-Gluconate, 140; *N*-(2-hydroxyethyl) piperazine-*N*′-ethanesulfonic acid (HEPES), 10; Na-Gluconate, 15; ethylene glycol-bis (2-aminoethyl)- *N*,*N*,*N*′,*N*′-tetraacetic acid (EGTA), 0.2; and NaCl, 4 (pH 7.2). Slices were incubated for 30 min at 35°C before being stored at room temperature in artificial CSF (ACSF) containing (in mM): NaCl, 125; NaHCO_3_, 25; KCl, 2.5; NaH_2_PO_4_, 1.25; MgCl_2_, 1; CaCl_2_, 2; and glucose, 25; bubbled with 95% O_2_ and 5% CO_2_.

Patch electrodes were pulled from hard borosilicate capillary glass (Sutter Instruments flaming/brown micropipette puller).

The AMPA/NMDA current ratios were measured in Mg^2+^-free ACSF. Postsynaptic currents were evoked by Schaffer collateral stimulation, while the CA1 pyramidal neurons were held at −70 mV. AMPA and NMDA receptor-mediated EPSCs were pharmacologically isolated by sequential bath application of APV and NBQX, respectively. First, compound AMPAR and NMDA-mediated current was recorded in Mg^2+^-free ASCF. After collecting at least 100 sweeps the AMPA-mediated component was blocked by application of 10 μM NBQX. Then, additional 100 sweeps of the putative NMDA-mediated currents were collected and the NMDA nature was confirmed by subsequent application of APV. The AMPA-mediated component was then obtained by subtraction of averaged the NMDA-mediated currents from the averaged compound response. For subsequent analysis, the mean amplitude of the AMPA currents was normalized to the amplitude of the NMDAR EPSCs.

The intracellular solution contained either 20 mM K2-ATP alone (PA-free), serving as the polyamine buffer, or was substituted with 100 μM of spermine (PA-containing). The free spermine concentration was ~7 μM.

For LTP recordings pipettes were filled with caesium-based solutions consisted (in mM) Cs-gluconate, 110; CsCl, 30; HEPES, 10; NaCl, 8; EGTA, 0.2; MgATP, 2; Na_3_GTP, 0.3 and phosphocreatine, 10 (pH 7.3 with CsOH). For measuring rectification of synaptic AMPARs pipettes were filled with either a polyamine-free (PA-free) or a polyamine-containing (PA-containing) solutions. The PA-free solution consisted of (in mM) Cs-gluconate, 100; CsCl, 10; HEPES, 10; NaCl, 8; EGTA, 0.2; MgATP, 2; Na_3_GTP, 0.3; phosphocreatine, 10; and K_2_ATP, 20 (pH 7.3 with CsOH). The PA-containing solution was identical except for the addition of 0.1 mM spermine, yielding a free spermine concentration of ~7 μM. Whole-cell voltage clamp recordings were made from CA1 pyramidal cells in 250 μm thick slices under visual control (Stuart et al., 1993). EPSCs were evoked from two independent inputs, one of which was paired and the other one was used as control, with two patch pipettes as stimulating electrodes. The two stimulus pipettes were >200 μm apart, located below the soma of a CA1 pyramidal cell. All measurements were at −70 mV membrane potential. LTP was evoked and recorded according to (Chen et al., 1999) by voltage clamping the membrane potential of the postsynaptic pyramidal cell to 0 mV for 3 min while stimulating the paired pathway every 1.5 s. Recording pipettes were filled with caesium-based solutions. The measured amplitudes were normalized to the mean EPSCs before pairing. For analysis, 5 subsequent responses were averaged. PhTx-433 (10 mM) was applied 40 min after starting the experiment to ensure washout of endogenous polyamines. The degree of blockade was calculated as a ratio of the average steady-state current amplitude before and after PhTx-433 application.

In experiments with synaptic IVs the command voltage was corrected for the liquid junction potential. Those cells, which still had detectable inward currents at 0 mV were not included in the analysis due to potential space clamp problems. Series resistance was monitored, and data from cells in which series resistance varied by >15% during recording were discarded from analysis. All experiments were done in the presence of SR-95531 to block GABAA receptor channels. Data are mean ±SEM; the statistical significance levels are from a Student's paired two-tailed *t* test for comparing responses from paired *vs*. unpaired input. LTP between genotypes was compared by ANOVA.

## Results

### Altered GluA2 distribution in CA1 pyramidal neurons of Homer1 KO mice

**Figure 1:**
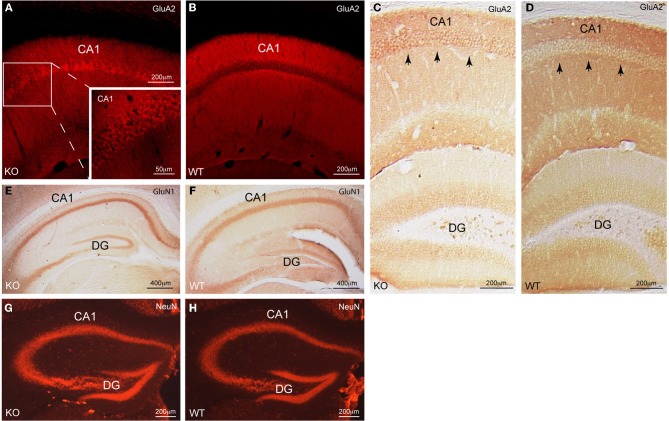
**Cellular distribution of GluA2 in CA1 pyramidal neurons of Homer1 KO mice. (A,B)** Immunofluorescent staining for GluA2 (red) on a saggital section of CA1 pyramidal neurons shows the differential distribution of GluA2 in Homer1 KO mice compared to WT littermates. Inset in A shows a close-up of the CA1 region and the enrichment of GluA2 immunoreactivity (red) in the pyramidal cell bodies (*n* = 6 mice/group). **(C,D)** DAB staining for GluA2 (dark brown) on a coronal section of the dorsal hippocampus shows the differential distribution of GluA2 in Homer1 KO mice compared to WT littermates. Black arrows point to pyramidal cell bodies in CA1 (*n* = 3 mice/group). **(E,F)** DAB staining for GluN1 (dark brown) on a coronal section of the dorsal hippocampus shows equal distribution of GluN1 in the pyramidal cell layer of Homer1 KO mice compared to WT littermates. (*n* = 3 mice/group). **(G,H)** Immunofluorescent staining for NeuN (red) on a coronal section, labeling neuronal cell bodies in the dorsal hippocampus of Homer1 KO and WT littermates (*n* = 3 mice/group). CA1, Cornu ammonis; DG, dentate gyrus, KO, knock-out; WT, wild-type.

We observed a significant redistribution of the AMPAR subunit GluA2 from the dendritic compartment into the cell bodies in CA1 pyramidal neurons of Homer1 KO but not WT mice (Figures 1A–D).

Note that the distribution of the GluN1 subunit of NMDARs (Figures 1E,F) as well as the distribution of neurons in dorsal hippocampus remained unchanged (Figures 1G,H). Thus, Homer1 proteins appear to selectively affect the subcellular distribution of GluA2-containing AMPARs in CA1 pyramidal neurons. To corroborate these findings we quantitatively assessed the distribution of GluA2 staining intensity in the pyramidal versus the dendritic layers of Homer1 KO and WT mice (see “Materials and Methods”). The ratios between the pixel intensity of a subfield (*x* = 300 μm, *y* = 120 μm) in the pyramidal cell layer compared to the underlying dendritic layer of Homer1 KO and WT mice (*n* = 3 confocal images from 3 individual mice/genotype) were 1.4, 1.2, 1.1 for Homer1 KO, and 0.8, 0.9, 0.9 for WT mice. Ratios above one clearly indicate stronger staining intensities in the pyramidal cell layer compared to the dendritic layer and thus a redistribution of GluA2 from the dendritic into the pyramidal cell layer in Homer1 KO mice.

### AMPA receptor properties in CA1 pyramidal neurons are regulated by Homer1 gene products

**Figure 2:**
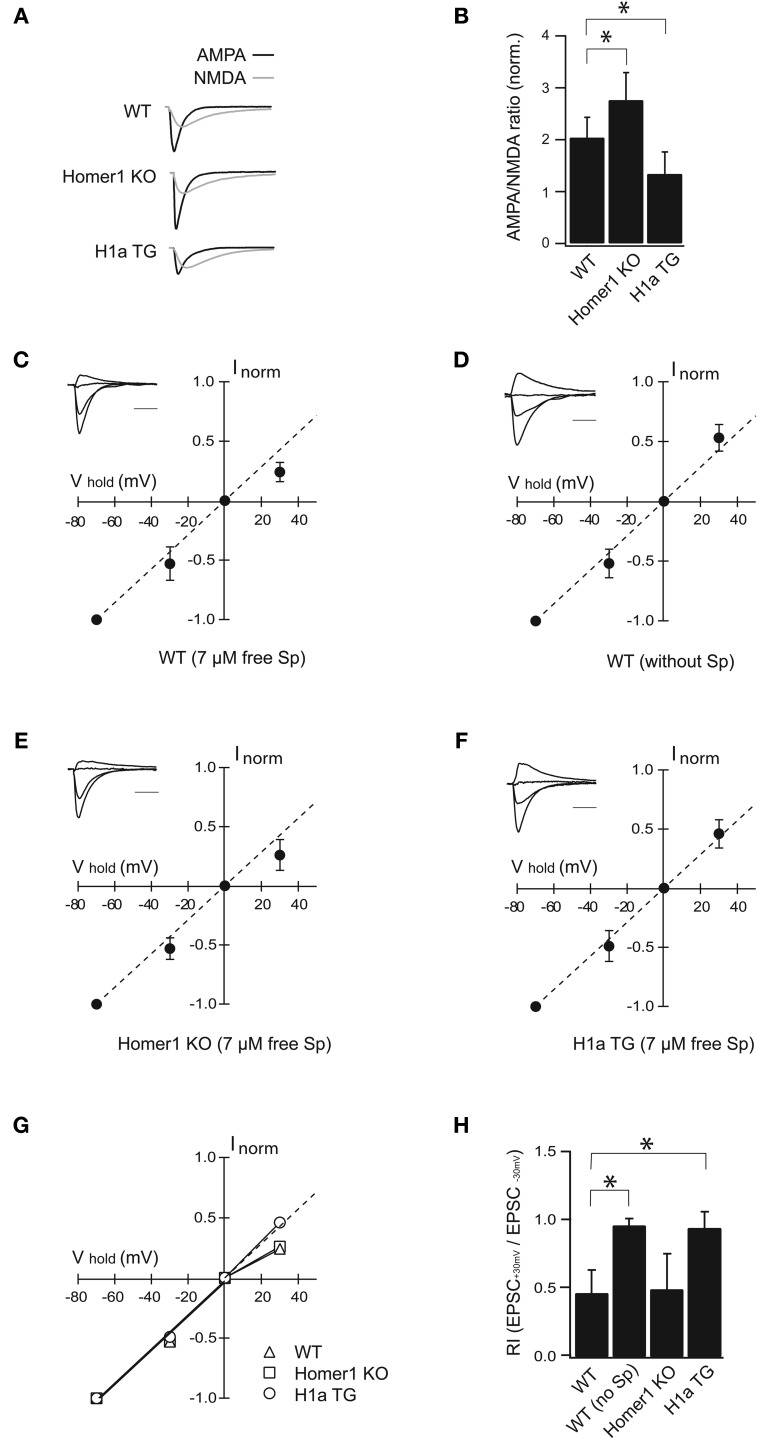
**H1a/Homer1 ratio alters the properties of AMPAR channels in CA1 pyramidal neurons. (A)** Averaged normalized traces recorded from CA1 pyramidal neurons after extracellular Schaffer collateral stimulation show evoked synaptic AMPA (black) and NMDA (gray) currents in wild-type (WT), Homer1 knock-out (Homer1 KO) and H1aTG mice. Scale bar: 40 ms. Stimulus artefacts have been omitted for clarity. **(B)** Histogram represents summarized data of AMPA/NMDA current ratios showing a strong reduction in H1a TG mice compared to WT. A significant enhancement of the AMPA/NMDA current ratio is observed in Homer1 KO mice compared to WT. **(C–F)** *I-V* plots show loss of polyamine sensitivity of synaptic AMPAR-mediated currents in H1a TG **(F)**, in comparison to WT **(C)**. Measurements from Homer1 KO mice **(E)** did not show any change in polyamine sensitivity. *I-V* plot in panel **(D)** shows experiments as described in **(C)**, but with polyamine-free intracellular solution. Recordings were performed from CA1 pyramidal neurons. Current amplitudes were measured at −70 mV, −30 mV, 0 mV, and +30 mV, respectively. Currents were normalized to the averaged amplitude of the response obtained at −70 mV. Insets represent sample traces for the given condition. The panel **(G)** shows summary plots of the *I–V* relationships from WT, Homer1 KO and H1a TG mice: the solid lines represent the linear fit to data points between the −70 and 0 mV holding potentials. The dotted line extends this fit to highlight the deviation of the *I–V* relationship from linearity. Bar histogram **(H)** shows rectification index (RI) expressed as EPSC_+30 mV_/EPSC_−30 mV_ obtained from WT, WT in PA-free condition, Homer1 KO and H1a TG mice, respectively. Error bars are SD, ^*^p < 0.05; Sp: spermine. Scale bar 20 ms; amplitudes are normalized.

To evaluate the effects of Homer1 and Homer1a on distribution of synaptic AMPARs we performed whole-cell voltage clamp recordings in CA1 pyramidal neurons and compared AMPA/NMDA current ratios in CA1 neurons of Homer1 KO, H1aTG and WT mice (Figures 2A,B).

Additionally, we tested whether the redistribution of GluA2 potentially affects the polyamine sensitivity of synaptic AMPARs by measuring the rectification indexes of AMPA-mediated EPSCs in WT, Homer1 KO, and H1aTG mice (Figures 2C–H).

Evoked synaptic AMPA receptor-mediated EPSCs measured from Homer1 KO mice were significantly enhanced compared to WT (AMPA/NMDA ratio 2.76 **±** 0.73 in Homer1 KO, *n* = 11 cells from 5 mice; *p* < 0.05; 2.04 ± 0.39 in WT, *n* = 17 cells from 7 mice; Figure 2B). In contrast, evoked synaptic AMPAR-mediated currents were strongly reduced in H1aTG mice in comparison to WT (Figures 2A,B), as indicated by the significantly smaller AMPA/NMDA current ratio (1.34 ± 0.42 in H1aTG, *n* = 11 cells from 3 mice; *p* < 0.01; Figure 2B).

AMPARs lacking the Q/R site-edited GluA2 subunit show marked inward rectification due to the voltage-dependent block of the channel pore by polyamines at positive membrane potentials (Hollmann et al., 1991; Burnashev et al., 1992; Jonas and Burnashev, 1995; Isaac et al., 2007). To examine changes in GluA2 subunit content, we tested the spermine sensitivity of synaptic AMPARs in the aforementioned different genotypes. EPSCs from CA1 pyramidal neurons were measured in whole-cell configuration at different holding potentials in either PA-free, or PA-containing intracellular solutions (Figures 2C,D). The rectification index (RI) was calculated as the EPSC_+30 mV_/EPSC_−30 mV_ amplitude ratio. When cells were patched with PA-containing solution, the evoked AMPAR currents in H1a TG mice did not rectify (RI = 0.95 ± 0.12; *n* = 10 cells from 6 mice), when compared to those in WT mice (RI = 0.47 ± 0.17; *n* = 14 cells from 6 mice; *p* < 0.01) (Figures 2C,F,G,H). Rectification properties of synaptic AMPAR channels from CA1 pyramidal neurons in Homer1 KO mice were comparable to those in WT mice (RI = 0.50 ± 0.26; *n* = 8 cells from 3 mice; *p* = 0.64; Figures 2C,E,G,H). To confirm that the observed inward rectification is indeed due to the polyamine block of AMPARs, we tested if washout of endogenous polyamines would lead to a linearization of the synaptic IVs. We ensured washout of polyamines from CA1 pyramidal cells in WT mice by prolonged whole-cell dialysis with PA-free, intracellular solution for 20 min before measuring of synaptic IVs. Washout of polyamines from pyramidal neurons resulted in a linear synaptic IV and a RI close to 1 (RI = 0.97 ± 0.05 mean ± SD; *n* = 7 cells from 4 mice; *p* < 0.01 compare to PA-containing solution; Figures 2D,H).

**Figure A1:**
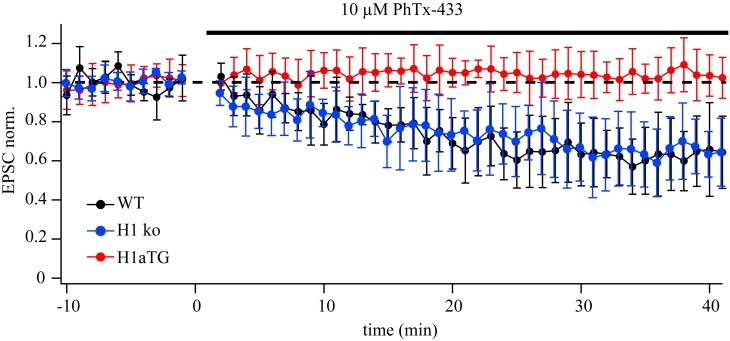
**Differential effect of PhTx-433 on AMPAR-mediated EPSCs in CA1 pyramidal cells of H1aTG and WT mice**. The time course of EPSC amplitude changes during PhTx-433 (10 mM) application in WT (black circles), H1 KO (blue circles) and H1aTG (red circles). PhTx was applied after achieving stable base line recordings. In WT as well as H1 KO slices, PhTx caused a significant reduction of EPSC amplitudes, (0.69 ± 0.17 *n* = 7 and 0.62 ± 0.16, *n* = 5, respectively) which reached steady state level 30 min after starting drug perfusion. In CA1 pyramidal cells of H1aTG mice PhTx did not have any significant effect on AMPAR-mediated synaptic transmission (1.02 ± 0.12, *n* = 5).

GluA2-lacking channels are blocked in an activity-dependent manner by PhTx-433, while GluA2-containing AMPARs are insensitive to the drug (Washburn and Dingledine, 1996; Rozov et al., 2012). To substantiate our finding that overexpression of Homer1a can decrease the contribution of synaptic GluA2-lacking channels, we compared the effect of PhTx-433 on EPSCs evoked in synapses from CA1 pyramidal neurons of WT, Homer1 KO, and H1aTG mice. We used a PA-free intracellular solution to exclude a possible effect of endogenous polyamines on the accuracy of the assay (Figure A1). While the application of PhTx-433 significantly reduced EPSC amplitudes relative to control values in WT (0.69 ± 0.17, *n* = 7, *p* < 0.05) and Homer1 KO neurons (0.62 ± 0.16, *n* = 5, *p* < 0.05), it did not affect the size of AMPAR-mediated EPSCs in CA1 neurons of H1a TG mice (1.02 ± 0.12, *n* = 5, *p* > 0.05) (Figure A1).

In summary these results demonstrate that Homer1a overexpression leads to a strong reduction in the amplitude of synaptic AMPAR-mediated currents, a decreased polyamine sensitivity of synaptic AMPARs and, hence, decrease the contribution of GluA2-lacking AMPARs to synaptic responses.

### Sustained H1a expression impairs LTP maintenance in hippocampal CA3-CA1 synapses

**Figure 3:**
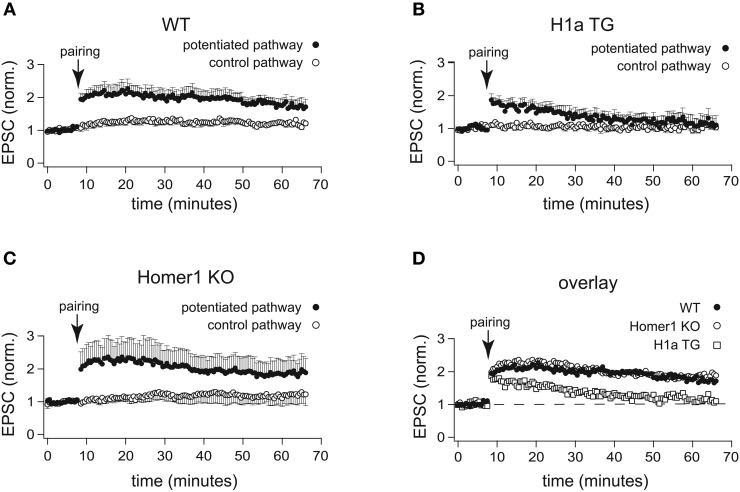
**H1a expression alters the maintenance of CA3-CA1 LTP in pyramidal CA1 neurons.** In comparison to the normalized amplitudes of the EPSCs of WT mice **(A)**, the amplitudes of EPSCs recorded from H1a TG mice **(B)**, after the initial potentiation, declined to the baseline levels within 20 min in the two-pathway LTP experiment, using the “pairing” protocol for induction. The analysis of LTP experiments obtained from Homer1 KO mice **(C)** revealed no differences when compared to WT, using “pairing” as an induction protocol. Closed circles: potentiated pathway; open circles: control pathway. Panel **(D)** represents an overlay of the potentiated pathways from WT (closed circles), Homer1 KO (open circles) and H1a TG (open squares) mice, using “pairing” as LTP induction protocol. Error bars are SEM.

Activity-dependent expression of H1a is involved in synaptic plasticity and memory consolidation (Koh et al., 2005). To investigate the effects of differential Homer1 gene expression in synaptic plasticity, we analyzed the amplitudes and time course of LTP at CA3-CA1 synapses (Figure 3).

Recordings from hippocampal CA1 pyramidal neurons were performed in whole-cell voltage clamp configuration. During pre- and post-induction phase of the experiments, cells were held at −70 mV. LTP was induced by pairing low frequency presynaptic stimulation of Schaffer collateral afferents (0.67 Hz) with postsynaptic depolarization to 0 mV. Clearly, “pairing” caused robust enhancement of the EPSC amplitudes in CA1 neurons of WT mice (2.00 ± 0.25 fold increase of the baseline measured 30 min after pairing; *n* = 7 cells from 5 mice; Figures 3A,D). In CA1 neurons of Homer1 KO mice LTP induction resulted in a comparable potentiation of the paired pathway (1.94 ± 0.46 fold increase of the baseline measured 30 min after pairing; *n* = 6 cells from 3 mice; *p* = 0.99; Figures 3C,D). In contrast, CA1 neurons of H1a TGs showed a transient potentiation of the paired pathway that decayed to baseline 20–30 min after induction (1.25 ± 0.17 fold increase of the baseline measured 30 min after pairing; *n* = 11 cells from 8 mice; *p* < 0.05; Figures 3B,D). These experiments suggest that the absence of all Homer1 isoforms has no effect on LTP induction and maintenance, whereas constitutively expressed short H1a impairs LTP maintenance.

### The effect of H1a on LTP depends on the presence of long Homer1 isoforms and GluA2

**Figure 4:**
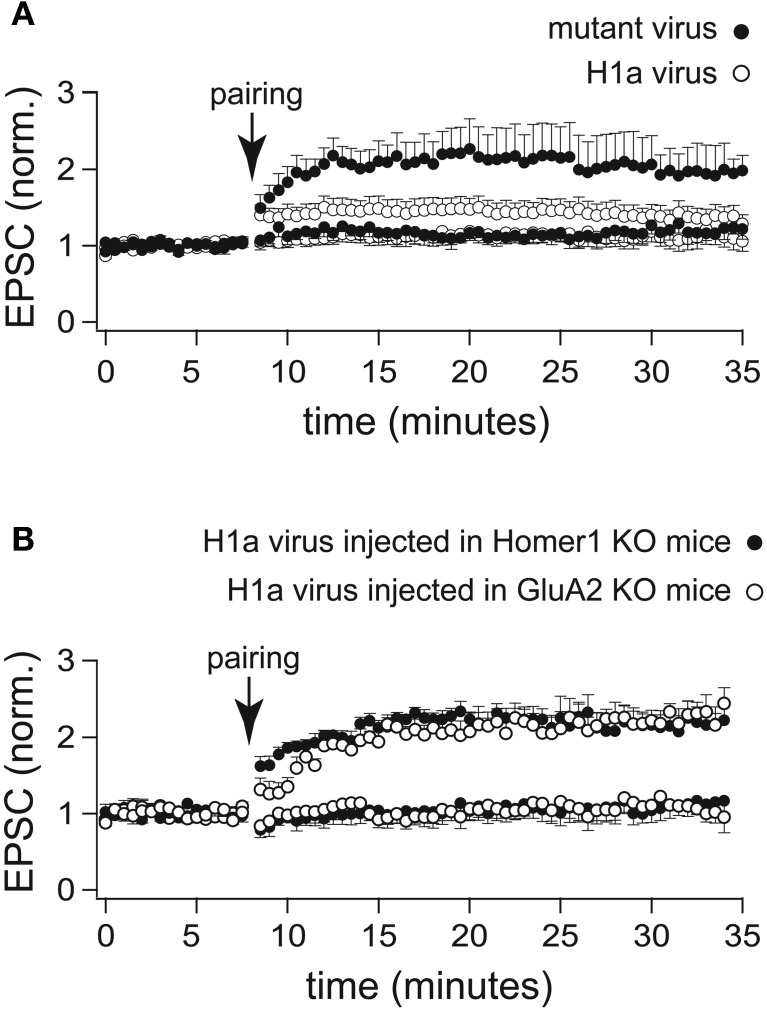
**LTP profiles recorded from CA1 pyramidal neurons infected with a lentivirus expressing H1a-Venus. (A)** Two-pathway LTP induced by the “pairing” protocol in CA3-CA1 synapses of WT mice stereotaxically injected with a lentivirus expressing H1a-Venus (H1aV) resulted in a transient potentiation of synaptic responses recorded from infected pyramidal neurons (open circles) of mutated H1aV (W234A) after lentivirus injection in WT mice showed an unaltered LTP profile of the paired pathway (closed circles). **(B)** Cellular LTP induced by the “pairing” protocol in either Homer1 KO (closed circles), or GluA2 KO (open circles) mice stereotaxically injected with lentivirus expressing H1aV revealed a comparable, stable potentiation of the paired pathway when recorded from infected, fluorescent cells. Error bars are SEM.

To identify the constituents essential for inhibition of LTP maintenance by H1a, we injected stereotaxically into the hippocampus of WT, Homer1 KO, and GluA2 KO mice (P42) recombinant lentivirus expressing either a point-mutated H1aV(W24A) or the wild-type form (Beneken et al., 2000; Celikel et al., 2007). The point mutation in H1aV substituted a tryptophane (W) with an alanine (A) in the EVH1 domain, thereby abolishing binding to proline-rich target sequences (Beneken et al., 2000; Celikel et al., 2007). Subsequently cellular LTP was measured from the infected, fluorescently labeled neurons 18 days post-injection (Figure 4A) (Celikel et al., 2007).

**Figure 5:**
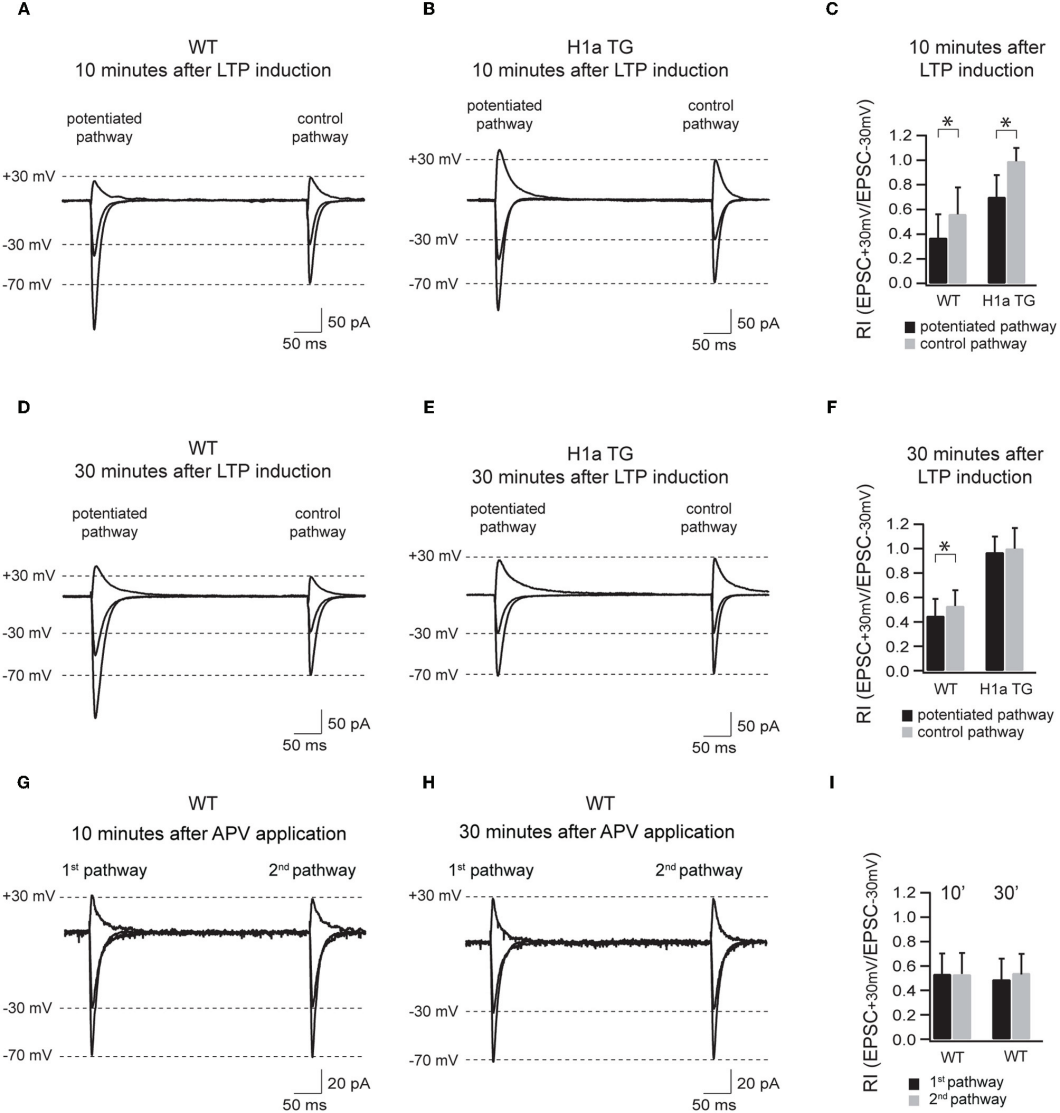
**Sustained H1a expression modifies the activity dependent incorporation of GluA2 subunit containing synapses.** Panels **(A–F)** show the AMPAR-mediated currents of the potentiated and control pathway 10 min after LTP induction (the “pairing” protocol) recorded from WT and H1aTG mice. Bar histogram **(C)** shows the summary of the rectification index (RI_+30 mV/−30 mV_) measured 10 min after LTP induction. Panels **(D–F)** show the same recording conditions performed 30 min after LTP induction. Note the constitutive expression of non-rectifying AMPARs both 10 min **(B)** and 30 min **(E)** after LTP induction measured from H1a TG mice. Panels **(G–I)** show the AMPAR-mediated currents of the two non-potentiated pathways recorded in WT within 10 min **(G)** and 30 min **(H)** after APV application. Bar histogram **(I)** shows the summary of the rectification indices (RI_+30 mV/−30 mV_) measured at 10 and 30 min, respectively. Shown traces represent the overlay of the averaged evoked EPSCs recorded at −70 mV, −30 mV, and +30 mV, respectively. Stimulus artefacts have been omitted for clarity. Error bars are SD, ^*^p < 0.05.

Virus-mediated overexpression of H1aV in CA1 pyramidal neurons of WT mice affected the induction and maintenance of LTP induced by the pairing protocol (1.26 ± 0.13 fold increase of the baseline measured 30 min after pairing; *n* = 12 cells from 5 mice; *p* < 0.05; Figure 4A). LTP maintenance was unaffected (2.01 ± 0.45; *n* = 7 cells from 3 mice; Figure 5A) in WT mice injected with the mutant H1a(W24A)V version.

We next stereotaxically injected recombinant H1aV expressing lentivirus in the hippocampus of Homer1 KO and GluA2 KO mice. H1a overexpression in these mouse lines did not affect the induction and maintenance of LTP in CA1 pyramidal neurons (Homer1 KO: 2.17 ± 0.20 fold baseline increase 30 min after pairing; *n* = 5 cells from 3 mice; GluA2 KO: 2.22 ± 0.18; *n* = 5 cells from 3 mice; *p* = 0.94; data are presented as mean ± SEM; Figure 4B) indicating that suppression of LTP requires a functional H1a EVH domain and both, functional expression of long Homer1 isoforms as well as GluA2.

### H1a modulates the activity-dependent incorporation of GluA2-containing AMPAR

The transient incorporation of GluA2-containing and GluA2-lacking AMPARs into synapses has been suggested to play an important role in LTP/LTD induction and maintenance (Liu and Cull-Candy, 2002; Bagal et al., 2005; Bellone and Luscher, 2006; Clem and Barth, 2006; Plant et al., 2006). To test if different Homer1 isoforms might be involved in the activity-dependent regulation of the synaptic AMPAR composition underlying long-term plasticity, we compared the polyamine sensitivity of synaptic AMPAR channels in potentiated and control pathways in CA1 pyramidal neurons of WT and H1aTG mice (Figure 5).

Based on these results we suggest that activity-induced expression of H1a is important for the activity-dependent incorporation of GluA2-containing AMPARs in synapses during LTP and therefore plays a crucial role in shaping hippocampal excitatory synaptic transmission.

## Discussion

Our data show that the IEG Homer1a can tune synaptic plasticity in excitatory pyramidal CA1 neurons by regulating the synaptic distribution of GluA2-containing AMPARs. Sustained overexpression of Homer1a reduced the polyamine sensitivity of synaptic AMPARs, suggesting an increase in the proportion of GluA2-containing synaptic receptors and leading to a reduction of averaged synaptic AMPAR-mediated conductance and AMPA/NMDA current ratios. However, the most prominent effect of sustained Homer1a expression is the loss of Schaffer collateral LTP.

Classically, Homer proteins represent molecular scaffolds linking cell surface constituents to internal Ca^2+^ stores, thus regulating intracellular Ca^2+^ concentration (Fagni et al., 2002; Huang et al., 2007; Worley et al., 2007; Feng et al., 2008). The IEG-form Homer1a is believed to uncouple the plasma membrane from internal Ca^2+^ stores, resulting in a blunting of synaptic Ca^2+^ signaling (Xiao et al., 2000). It is, however, unlikely that Homer function in store-released Ca^2+^ signaling is important for LTP establishment, since LTP is not compromised in neurons of Homer1 knock-out mice and, more importantly, in Homer1 KO mice overexpressing Homer1a. Even if Homer2/3 would still allow the physical linkage between plasma membrane and intracellular Ca^2+^ stores, overexpression of Homer1a should result in uncoupling, due to the high conservation of the N-terminal EVH domain and thus the loss of LTP. Furthermore, knock-out of receptors for intracellular Ca^2+^ release associated with Homer isoforms, the IP3-R and RyR, does affect LTP maintenance (Fujii et al., 2000; Shimuta et al., 2001).

The most intriguing aspect of the data presented here is the strict dependence, as revealed by the RIs, of the Homer1a-mediated suppression of LTP on the synaptic expression of GluA2-containing AMPARs. Although the RI significantly decreased in the potentiated pathway when compared to the control pathway 10 min after LTP induction, the RI was comparable in both pathways 30 min after LTP induction, indicating that the vast majority of synaptic AMPARs were constituted by GluA2-containing receptors. Notably, at the same time, EPSC amplitudes in the potentiated pathway declined to the values observed prior to potentiation.

In WT neurons, the decrease in rectification between potentiated and control pathway was less pronounced, but remained significant, indicating an enhanced contribution of GluA2-lacking AMPAR channels. In this respect this finding supports a notion proposed by Plant et al. (Plant et al., 2006) that GluA2-lacking AMPARs are involved in LTP induction. Our data clearly show that these channels also contribute to the later stages of LTP. Probably, absence of synaptic GluA2-lacking AMPARs in H1aTG mice affects the maintenance phase of LTP and results in a fast reduction of EPSC amplitudes following pairing.

However, there is an apparent contradiction between the data reported by Plant and co-workers and our results. In contrast to our findings Plant et al. showed a strong and significant reduction of polyamine sensitivity 30 min after LTP induction. This might be due to differences between the two protocols used to measure RIs. Plant et al. did not block the NMDA component for an obvious reason: wash-in and subsequent wash-out of APV to measure the RIs during base line drastically increases the time of whole-cell dialysis prior to LTP induction and thus would lead to a “washout” of LTP. Instead they took another approach and assumed that NMDAR-mediated currents remain unaffected by LTP induction protocols. Thus, for RIs quantification they used compound AMPAR and NMDAR mediated responses. While at negative potentials NMDARs are strongly blocked by Mg^2+^ and therefore do not contribute significantly to the EPCS amplitudes, at +30 mV NMDAR input to the amplitude of compound EPSCs is significant. Most of the LTP induction protocols are designed to provide robust and long lasting elevation of postsynaptic Ca^2+^, however, intracellular Ca^2+^ blocks NMDARs (Medina et al., 1996). Consequently, the contribution of NMDARs to the compound response is reduced right after LTP induction. This reduction is more obvious at positive potentials. However, at later time points the postsynaptic Ca^2+^ concentration decreases to basal levels and the NMDAR channel block is relieved. Thus, an enhancement of outward currents at 30 min post induction might be due to an increased contribution of NMDARs. On the other hand, we also showed a slight reduction of AMPAR rectification at later time points. Moreover LTP can be evoked in GluA1 KO mice, but detectable response enhancement is delayed by 40–60 min compared to WT mice (Hoffman et al., 2002). Thus, GluA1-lacking (most likely GluA2-containing) channels are likely to contribute to the maintenance of LTP. It has been suggested previously that LTP may be substantially increased in mice with GluA2-lacking AMPARs (Jia et al., 1996; Meng et al., 2003). However, in our study we used the GluA2 KO mice line analyzed by Feldmeyer et al. (Feldmeyer et al., 1999) with no significant change in LTP level as compared to WT littermates.

Numerous studies indicate a crucial role of GluA2-lacking channels during the initial steps of LTP induction (Plant et al., 2006; Moult et al., 2010). There is also convincing evidence for the GluA1 subunit as a key player for LTP generation; deletion of the GluA1 gene in mice abolishes LTP at Schaffer collateral synapses (Zamanillo et al., 1999). Moreover, LTP is suppressed in mice expressing GluA1 only in a Q/R site-edited version (in preparation). Thus, not only is GluA1 essential for LTP induction, but also its ability to form high-conductance AMPA channels. Moreover, GluA2-containing channels are involved in LTD induction (Liu and Cull-Candy, 2002), and some forms of LTD are absent in GluA2 KO mice. Thus, we suggest that the synaptic ratio between GluA2-containing and GluA2-lacking (GluA1-containing) AMPA channels determines the probability of up-regulating (LTP) or downsizing (LTD) transmission at a given synapse. Collectively, our hypothesis is based on the findings that (1) the subcellular distribution of the GluA2 subunit depends on the expression of long Homer1 isoforms; (2) the expression of Homer1a triggers the substitution of synaptic GluA2-lacking by GluA2-containing AMPARs (perhaps by interrupting the interaction between long Homer1 and extrasynaptic GluA2-containing channels) and (3) the expression of both, long Homer1 isoforms and GluA2 subunits is required for the Homer1a effect. In this respect, the phenomenon described in this study suggests a novel activity-dependent mechanism controlling synaptic AMPAR subunit composition and, therefore, synaptic function.

### Conflict of interest statement

The authors declare that the research was conducted in the absence of any commercial or financial relationships that could be construed as a potential conflict of interest.
